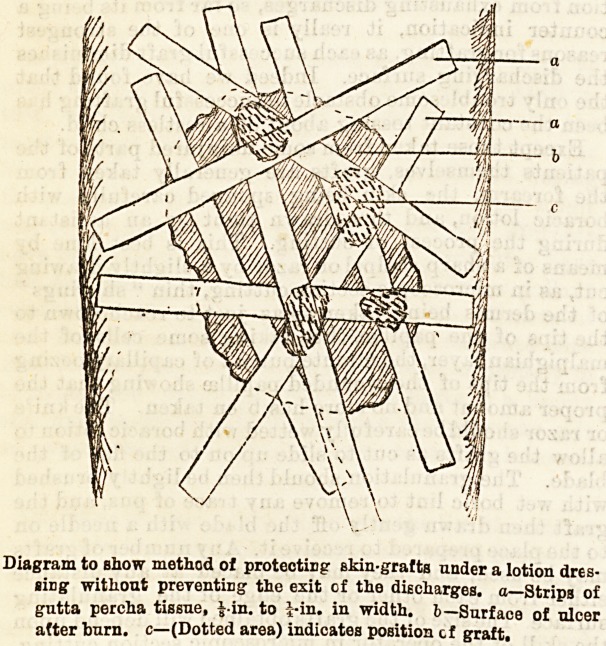# Treatment of Burns and Scalds

**Published:** 1892-10-22

**Authors:** 


### The Hospital Clinic.

[*The Editor will be glad to receive offers of co-operation and contributions from members of the profession. All letters should be addressed to* The Editor, The Lodge, Porchester Square, London, W.]

### ROYAL FREE HOSPITAL.

### Treatment of Burns and Scalds.

Since burns and scalds form such a large and important proportion of the accidents that occur in the practice of both hospital surgeons and general practitioners, the following sketch of the general principles of treatment, based upon an account of the practice of the Royal Free Hospital, will be of interest:—

If the case is at all serious the patient is at once carried to bed without waiting to make any examination of the injuries, or to remove charred clothing until all preparations for the dressing have been made—these include the administration of some stimulant, such as brandy diluted with about two parts warm water, in quantity suited to the age of the patient. Hypodermic injections of ether may be useful in urgent cases of shock, but whatever may be given should be given in sufficient quantity to have a marked stimulant effect, and should be repeated, especially during the process of dressing the injuries, should the pulse show sign of heart failure. Tincture of opium (♏ xx or xxx for an adult) should be given with the stimulant when pain is severe, except in the case of infants, for whom a few grains of bromide of potassium are safer, and appear to act quite as well. Charred clothing should be removed with as little disturbance of the patient as possible, only a small part of the injured surface being exposed at a time, and a dressing at once applied to this. For the first twenty-four hours a dressing of lint soaked in "carron oil" (lime water and linseed oil, equal parts) is found most useful as a sedative to the pain. Over this is laid some oil-paper, and a thick layer of cotton wool fixed on with bandages, care being taken not to apply these more tightly than is absolutely necessary. This dressing should be left undisturbed for twenty-four hours if possible, but where there is much discharge, and especially in the case of very restless children, it may require changing after twelve hours. Bathing an injured limb in warm boric acid lotion (gr. xv ad. ℥i) often assists the removal of charred clothing and separated epidermis, and in the case of children with extensive burns a warm boric lotion bath is useful. This has been also recommended for adults, but is seldom practicable without an amount of disturbance of the patient that does more harm than the bath does good. After the first twenty-four hours it is customary to change the carron oil dressing for some antiseptic ointment; perfect asepsis is practically impossible, but eucalyptus ointment (1 in 15) has been found most useful at the Royal Free Hospital. The loose skin over the blisters may now be carefully removed, as if left it only forms pockets for the accumulation of septic discharges. It is not often necessary to change the eucalyptus dressing more than once or twice in twenty-four hours, and this treatment is usually continued until any sloughs that may form are beginning to separate when a lotion dressing changed every four or six hours is often very useful. Lint soaked in boric or chlorinated soda lotion (lig. sodæ chlor. ʒi, aq. ℥ii) is generally used, the latter especially where the sloughs are large and the discharge offensive, or if the granulations are indolent; over the lint is laid the usual cover of oil-paper or gutter-percha tissue, with a good layer of cotton wool to retain the moisture of the dressing. For this purpose also the addition of a little glycerine to the lotion is useful (℥i, ad. Oj.)

When the sloughs have separated, the granulating surface left may be dressed with any simple lotion or ointment dressing until healed; but attention must be paid to hastening the healing as the best preventive of serious cicatricial contraction causing deformity. For this, the practice of skin grafting by a modification of Thiersch's method has been found very helpful. The surface of the ulcers must be carefully cleansed and prepared for the grafts by a dressing of warm boric lotion, changed every three or four hours for at least twelve hours before the grafting, but it is better not to exceed twenty-four hours, as then the granulations tend to become somewhat flabby and œdematous. With regard to the condition of the patient as affecting the success of skin grafting, we may remark that this appears to be of much less importance than has generally been stated, for we have observed that grafts have "taken" perfectly well as soon as ever a granulating surface has been formed, in spite of the presence of febrile temperature, in one case ranging from 101 deg. to 104 deg., and even of active secondary syphilis, some of the successful grafts in the latter cases being taken from the patients themselves. With regard to emaciation from exhausting discharges, so far from its being a counter indication, it really is one of the strongest reasons for grafting, as each successful graft diminishes the discharging surface. Indeed we have found that the only troublesome obstacle to successful grafting has been the constant tossing about of a restless child.

Except those taken from some uninjured parts of the patients themselves, grafts are generally taken from the forearm, the skin being sponged carefully with boracic lotion, and then drawn tight by an assistant during the process of cutting. This is best done by means of a sharp scalpel or razor by a slightly drawing cut, as in microscopic section cutting, thin "shavings" of the dermis being taken so as just to reach down to the tips of the papillæ, thus taking some cells of the malpighian layer, the minute puncta of capillary oozing from the tips of the wounded papillæ showing that the proper amount and no more has been taken. The knife or razor should be carefully wetted with boracic lotion to allow the grafts as cut to slide up on to the flat of the blade. The granulation should then be lightly brushed with wet boric lint to remove any trace of pus, and the graft then drawn gently off the blade with a needle on to the place prepared to receive it. Any number of grafts may be used, and they may be placed at any distance either from each other or the edge of the granulating surface. The size of the grafts obtained will depend upon the skill of the operator in microscopic section cutting, also upon the generosity of the donors of the grafts, for whose benefit we may remark that as the epithelium is only partially removed, the places soon heal over and cause no trouble at all after the first day. A small piece of dry absorbent wool laid on forms a firm scab with the slight capillary oozing from the wounded tips of the papillæ, under which healing takes place quietly, a faint scar being left, which is practically imperceptible after a few weeks. Further, the process of cutting if the knife is sharp, and the skin held steadily and stretched, is hardly felt at all. The most successful grafts we ever saw were taken in pieces, ranging up to one inch in diameter, from an amputated limb directly after removal. They were applied so as to cover up the raw surface on a baby miserably emaciated with the discharge from an extensive burn on the abdomen; every graft took, and in a few days the whole was healed up.

After the grafts are laid on it is necessary to protect them from being removed at the change of dressing, and this can be done by a series of strips of gutta percha laid across the wound in various directions, crossing over the grafts, and fixed down to the healthy skin around by the application of a trace of chloroform

(see diagram). Over this is laid a four or six-fold layer of lint, soaked in warm boric acid lotion, covered entirely with oil paper or sheet gutta percha tissue and wool to keep in the moisture. This dressing should be changed every six hours for two days, care being taken not to disturb the gutta percha strips, or at any rate to replace them if moved. In this way the grafts are anchored in position until they have had time to "take root," and the discharge from the uncovered granulations can escape freely into the dressing. After the first four days it is generally possible to go back to the ordinary ointment dressing, as the grafts will be sufficiently secure, unless more grafting is required.* Should cicatricial contraction lead to serious deformity, plastic operations may be necessary later, but space does not permit us to consider these now. Suffice it to say, that rapid healing over is the most important thing, and gentle passive motion with each dressing will often save a good deal in the case of burns at the flexures of the limbs.

With regard to the constitutional treatment, there is little to be said, except that during the first few days

Diagram to show method of protecting skin-grafts under a lotion dressing without preventing the exit of the discharges. *a*—Strips of gutta percha tissue, $\frac{1}{8}$-in. to $\frac{1}{4}$-in. in width. *b*—Surface of ulcer after burn. *c*—(Dotted area) indicates position of graft.

only the simplest food should be given, such as milk, milk and soda water, beef tea, or barley water, with some of the farinaceous foods specially prepared for invalids. The possibility of gastro-intestinal trouble should be borne in mind, though it is apparently not so frequent now owing to the means used to diminish the septicity of the discharges from the burns. Stimulants during the period of shock are certainly valuable, combined with opium or some other sedative, if necessary, to secure sleep, or, at any rate, limit restlessness. During the process of separation of the slough and subsequent healing the exhaustive effect of the discharges must be combated by liberal feeding, and everything should be done to encourage the appetite bya varied, though simple, diet; and a little stimulant, in the form of wine, beer, or stout, given with meals, is often of great value as an aid to digestion.

* In the diagram only a few small grafts are shown for the sake of clearness: in practice, the whole surface should be covered if sufficient grafts can be obtained, the intervals between the strips of gutta percha tissue being sufficient to allow the escape of any discharge.

## Figures and Tables

**Figure f1:**